# Experiences of breast cancer in Arab countries. A thematic synthesis

**DOI:** 10.1007/s11136-019-02328-0

**Published:** 2019-10-23

**Authors:** D. Fearon, S. Hughes, S. G. Brearley

**Affiliations:** 1 International Observatory on End of Life Care, Faculty of Health and Medicine, Lancaster University, Lancaster, UK; 2 Cairdeas International Palliative Care Trust, Nouakchott, Mauritania

**Keywords:** Quality of life, Breast cancer, Arab, Muslim, Qualitative, Thematic synthesis

## Abstract

**Background:**

Breast cancer is the most common cancer in women globally. Its negative effects on a woman’s quality of life are related to the individual and socio-cultural factors. This review aimed to identify and synthesise the reported experiences and quality of life of women with breast cancer in Arab countries.

**Methods:**

PubMed, Embase, Web of Science, SCOPUS, PsychInfo, CINAHL, Allied and Complementary Medicine Database, and Index Medicus for the Eastern Mediterranean Region were searched for articles published from start to March 2019 using PRISMA guidelines. These searches were complimented by citation tracking and  hand searching of relevant journals. A thematic synthesis was carried out on the ‘findings/results’ sections from the identified papers.

**Results:**

Of 5228 records identified, 19 were included in the review which represented 401 women from 11 Arab countries. All used qualitative methods of data collection to produce rich descriptions of experiences. Thematic synthesis of the extracted data identified three major themes, Perceptions and reactions, Coping or enduring and Changing roles.

**Conclusions:**

This review provides a rich description of the reported quality of life and experiences of women with breast cancer in Arab countries. These are influenced by the women’s and society’s views of cancer, the women’s role in society and family, religious faith and the healthcare context and access to treatment choices and information.

**Electronic supplementary material:**

The online version of this article (10.1007/s11136-019-02328-0) contains supplementary material, which is available to authorised users.

## Background

Breast cancer is the most common cancer diagnosed in women globally [1]. High-income regions, such as North America and Europe, experience the highest rates of incidence, considered to be due to screening and earlier detection, and genetic differences [1, 2]. Inversely, its incidence is lowest but mortality highest in low- and middle-income regions, due to the limited diagnostic and treatment capacities [1, 3, 4]. In the Arab world, breast cancer ranks as the most frequently diagnosed cancer overall, representing an estimated 17.7 to 19% of all new cancers in 2018 [5, 6]. Its prognosis is relatively better than in much of sub-Saharan Africa. There is, however, much variation across the region, with worse outcomes seen in lower to middle-income countries; for example, the 5-year survival rates for breast cancer are 43.1% in Jordan compared with 85.3% in Qatar [7].

Research exploring the impact of breast cancer on women’s health-related quality of life (HRQOL) and experiences has explored how experiences change over time. The time of diagnosis is associated with shock and fear, feelings which fluctuate over the course of treatment and as the women face either progression or survivorship [8–10]. These experiences and the journeys are influenced by cultural and societal values. Much of this cultural understanding comes from research with ethnic minority groups in higher income regions; however, there is an increasing body of research from different regions, such as Arab contexts. Two recent reviews [11, 12] of HRQOL of women with breast cancer in the Arab region identified two common measurement scales, the European Organization for Research and Treatment in Cancer Quality of Life Questionnaire (EORTC QLQ-C30) and the EORTC breast cancer specific quality of life questionnaire (EORTC QLQ-BR23). These scales provide a measurement of global HRQOL, symptom burden, role functioning and breast-specific aspects of HRQOL. Haddou Rahou et al. [11] found much heterogeneity in the limited data originating from Arab countries, with a mean global HRQOL score ranging from 45.3, in Kuwait, to 74.6, in Bahrain, on a scale of 0 to 100, with 100 representing the best well-being. They subsequently performed a narrative analysis of the data rather than a meta-analysis concluding that Arab women have low quality of emotional well-being but higher levels of social well-being, which they suggest is due to the strong family links and support available. Hashemi et al. [12] more recently explored experiences in the Eastern Mediterranean region and found a similar broad range of global HRQOL scores, ranging from 31.1 in Saudi Arabia to 75.6 in Qatar. They also found lower scores for emotional well-being and higher scores for social functioning; however, their meta-analysis has limited utility for the Arab context because 19 out of the identified 36 articles originated from Iran or Pakistan, neither of which are Arab countries. These reviews, and the use of scales, are helpful to understand, measure and compare reported experiences and HRQOL of women with breast cancer. Such understanding can be enriched with data originating from qualitative methods of data collection, which provide deeper, richer understanding of individuals’ experiences of a phenomenon such as breast cancer [13]. Consequently, this review aimed to identify and synthesise the available literature exploring Arab women’s experiences of breast cancer from such a qualitative perspective. This was guided by the review question, ‘What is the experience of Arab women with breast cancer?’

## Methods

This review followed the Preferred Reporting Items for Systematic Reviews and Meta-Analyses (PRISMA) guidelines [14]. Data extraction, analysis and synthesis followed Thomas and Harden’s thematic synthesis approach, which translates the concept of thematic analysis of primary data to secondary data [15–17]. This approach aims to go beyond the studies authors’ original findings to produce new, higher-level understanding of the phenomenon of interest.

### Search strategy

The following electronic databases were searched from the earliest available date to March 2019, PubMed, Embase, Web of Science, SCOPUS, PsychInfo, CINAHL, Allied and Complementary Medicine Database, and Index Medicus for the Eastern Mediterranean Region. Search terms were truncated where appropriate (Table 1). The search strategy for PubMed can be found in the Supplementary Information. This broad range of terms was selected due to their relevance to the subject area and the relative difficulty of finding qualitative research [15]. The electronic database search was complemented with

- tracking of citations found in the identified articles and reviews;
- hand searching of the indexes of the following regional journals: The Middle East Journal of Family Medicine, Oman Medical Journal, Journal Medical Libanais, La Tunisie Médicale, the Arab Journal of Psychiatry and the Pan African Journal of Oncology;
- experts in the region were personally contacted for suggestions on eligible articles.

**Table 1:** Search terms used with electronic databases

Participant	Experience	Location
(Breast OR Mammary) AND (Cancer OR Tumour OR Tumor OR Malignancy OR Neoplasm)	Adaptation, attitudes, anxiety, barrier, belief, believe, coping, culture, depression, enduring, expectation, experience, health knowledge, idea, lived experience, motivation, narrative, perception, perspective, psychological, quality of life, social support, survivor, view	Arab, Algeria, Bahrain, Comoros, Djibouti, Egypt, Iraq, Jordan, Kuwait, Lebanon, Libya, Mauritania, Morocco, Oman, Palestine, Qatar, Saudi Arabia, Somalia, Sudan, Syria, Tunisia, United Arab Emirates, Yemen

### Selection criteria

Articles were included in the review if they met the inclusion and exclusion criteria in Table 2.

**Table 2:** Inclusion and exclusion criteria

	Inclusion	Exclusion
Study design	Original research with qualitative design published in peer-reviewed journals	Review articles, books, conference articles, posters, letters to editor and opinion pieces
Language	Published in English or French	Non-English, non-French language
Focus	Experiences of breast cancer	Other cancers or impossible to disaggregate data relating to breast cancer from other cancers
	Experiences of women with breast cancer	Experiences of relatives, healthcare professionals, or men with breast cancer
Context	Arab women in an Arab country	Immigrant Arab women in a non-Arab country

### Selection procedure

Identified items were imported into Papers for Mac version 3 [18] and duplicates were removed. Titles and abstracts were screened against the inclusion criteria by the author DF. Full-text articles were retrieved and assessed for articles meeting the inclusion criteria or when in doubt.

### Quality assessment

Quality assessment of included studies was not performed. Many established quality-assessment tools have limited utility in research from non-Anglophone countries, especially those with less established research platforms [19, 20]. Such tools tend to measure the quality of report writing rather than the identification of interesting and novel findings [21]. Finally, there is no established cut-off of quality below which the exclusion of papers would be justified or beneficial [16].

### Data extraction and synthesis

All papers were read and re-read before and during the analysis. Study characteristics, such as research aims, study design, sample size, participant demographics and main findings were extracted and tabulated. All data found under the headings ‘findings’ and/or ‘results’, including participant’s quotations, were exported verbatim into N-Vivo for Mac v.10.2.2. Analysis and synthesis of these data followed Thomas and Harden’s guidelines [15]. Extracts were read and re-read, and then coded line by line. Codes were then linked into clusters and subsequently into themes. Themes were examined for interconnectedness across the sample with the development of superordinate theme.

## Results

**Fig. 1:**
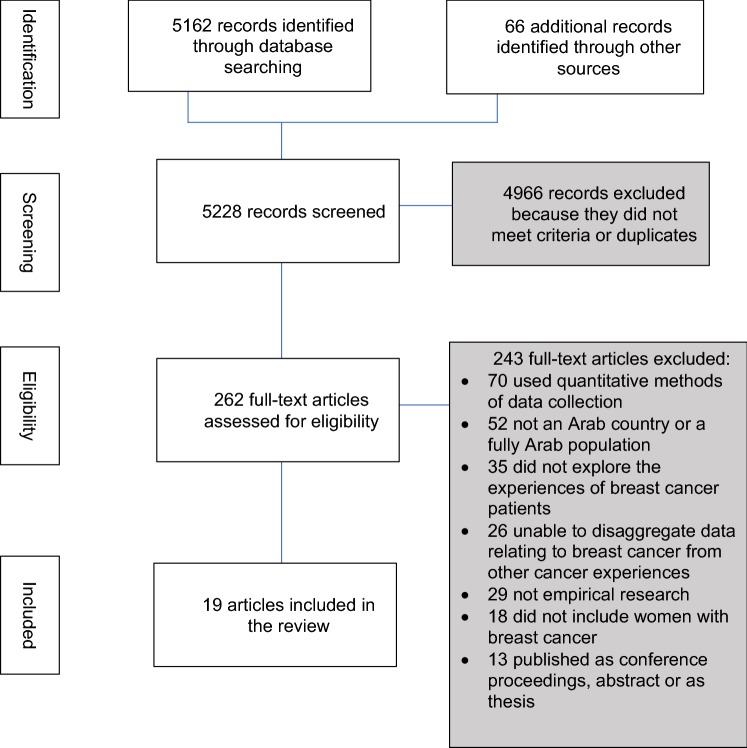
Flow diagram to show literature search process in accordance with PRISMA

A total of 5228 citations were identified, of which 262 papers were selected for full-text review after the removal of duplicates and the screening of titles and abstracts (Fig. 1).

### Study characteristics

The characteristics and main findings of the 19 included studies are displayed in Table 3. Sample size ranges from 2 to 60, with a total of 401 women with breast cancer from 11 Arab countries; Bahrain (*n* = 1), Egypt (*n* = 1), Jordan (*n* = 3), Lebanon (*n* = 4), Morocco (*n* = 1) Oman (*n* = 2), Palestine (*n* = 1), Syria (*n* = 1), Saudi Arabia (*n* = 2), Tunisia (*n* = 1) and United Arab Emirates (*n* = 2). Participants’ ages range from 24 to 71 years old. All disease stages are represented, and time since diagnosis ranges from 3 months to 9 years. Research aims include the impact of diagnosis, issues around late diagnosis, the challenges of active treatment, survivorship, familial and social support networks, sexuality, and access to information.

**Table 3:** Characteristics of the included studies

First author	Year	Country	Research focus	Methods	*n*	Demographics	Main findings
Al-Azri [22]	2014	Oman	Coping strategies after diagnosis	Semi-structured interviews (single). Framework analysis	19	Aged 24 to 54 years old All stages of disease and treatment Time since diagnosis: 3 months to 3 years (mean 12.2 months)	Six themes: (a) denial, (b) optimism, (c) withdrawal, (d) religious beliefs and practices, (e) support of family members and healthcare providers
Al-Azri [23]	2014	Oman	Psychosocial impact of diagnosis	Semi-structured interviews (single). Framework analysis	19	Aged 24 to 54 years old. Attending outpatients or hospitalised. All stages of disease and treatment Time since diagnosis: 3 months to 3 years (mean 12.2 months)	Four themes: (a) psychological distress of the disease and uncertainty, (b) reactions of family members, (c) views of society and (d) worries and threats about the future
Alqaissi [24]	2010	Jordan	Meanings of social support through the cancer journey	Semi-structured interviews (single). Heideggerian hermeneutical analysis	20	Aged 24 to 72 years old. All stages of disease. Late stage or post treatment Time since diagnosis: 6 to 24 months (mean 12.7 months)	Six themes: (a) stigmatised disease, (b) social support, (c) being strong for self and others, (d) resources, (e) controlling information for protection and (f) spiritual meaning as support One constitutive pattern: culture influencing the meaning of social support
Almegewly [25]	2019	Saudi Arabia	Perspectives of survivorship following treatment	Interviews (single). Interpretative phenomenological analysis	18	Aged 30 to 50 years old with stage I or II. 6 to 47 months post treatment	Three themes: (a) the meaning of cancer, (b) hidden survival and concealing the diagnosis and (c) cultural meanings of survival
Assaf [26]	2017	United Arab Emirates	Experiences following diagnosis and treatment	Semi-structured interviews (single)	20	Aged 30 to 65 years old. All disease stages. Undergoing active treatment. Time since diagnosis: 8 to 24 months	Three themes: (a) protecting oneself from stigma, (b) facing uncertainties and (c) getting on with life
Doumit [27]	2007	Lebanon	Lived experiences of oncology patients receiving palliative care	Semi-structured interviews (single). Phenomenological analysis (Utrecht School)	3	Age NR Palliative stages of disease Time since diagnosis: NR	Eight themes: (a) distress at being dependent, (b) dislike for pity, (c) worry for the family, (d) reliance on God, (e) dislike of hospitals, (f) fear of pain and (g) need to communicate
Doumit [28]	2010	Lebanon	Coping strategies	Semi-structured interviews (longitudinal). Seven-stage hermeneutical analysis (Diekelmann and Ironside)	10	Aged 36 to 63 years old. All disease and treatment stages. Time since diagnosis: 4 months to 9 years	Seven themes: (a) cancer is from God, (b) cancer is similar to any other disease, (c) positive support from others, (d) sharing the experience, (e) changed body image, (f) fear of reoccurrence and (g) pity Constitutive pattern: cancer is a cut in our lives that we have to fight
Doumit [29]	2010	Lebanon	Lived experiences of BC	Semi-structured interviews (multiple). Phenomenological analysis (Utrecht School)	10	Aged 36 to 63 years old. All disease and treatment stages. Time since diagnosis: 4 months to 9 years	Four themes: (a) living with losses, (b) living with guilt feeling, (c) living with fears and uncertainty and (d) living with a need to know
Elobaid [30]	2016	United Arab Emirates	Delayed presentation and health seeking behaviour	Semi-structured interviews (single). Thematic framework analysis	19	Aged 35 to 68 years old. Advanced stages of disease. Time since diagnosis: < 2 years	Three themes: (a) symptom recognition and appraisal, (b) role of community and social network and (c) healthcare delivery system
Hammoudeh [31]	2017	Palestine	Experiences of BC and its impact on families and social relationships	Semi-structured interviews (single) and focus group discussions. Thematic analysis	35	Aged 25 to 71 years old. All stages of disease and treatment. Time since diagnosis: < 1 year to several years	Three themes: (a) the transition from shock to the daily struggles, (b) the role of social support in helping women cope and (c) faith and reliance on God
Jassim [32]	2014	Bahrain	Experiences, beliefs, perceptions and attitudes relating to QOL in BC	Semi-structured interviews (single). Constant comparative analysis	12	Aged 39 to 68 years old. All stages of disease and treatment. Time since diagnosis: 1 to 8 years (mean 2.6 years)	Five themes: (a) meaning of cancer and QOL, (b) spirituality and beliefs about causes of breast cancer, (c) coping mechanisms, (d) impact of illness and (e) change in relationships
Kobeissi [33]	2014	Lebanon	Translation and validation of FACT-B into Arabic	Focus group discussions	41	Aged 20 to 71 years old. All stages of disease and treatment. Time since diagnosis: NR	Five themes: (a) physical well-being, (b) social/family well-being, (c) emotional well-being, (d) functional well-being and (e) additional concerns
McEwan [34]	2014	Egypt	Experiences of diagnosis and treatment delays	Semi-structured interviews (single). Thematic content analysis (social ecological model)	15	Aged 29 to 60 years old. All stages of treatment. Staging of disease: NR. Time since diagnosis: NR	Four themes: (a) intrapersonal factors, (b) interpersonal relationships, (c) institutional factors and (d) public policy factors
Masmoudi [35]	2016	Tunisia	Issues of sexuality	NR	2	Aged 44 to 48 years old. Post mastectomy. Time since diagnosis: NR	Five themes: (a) difficulties of communicating with the doctor, (b) erroneous beliefs, (c) reactive depression, (d) changing body image and (e) trouble communicating within the couple
Nizamli [36]	2011	Syria	Experiences of chemotherapy	Semi-structured interviews (single). Content analysis	17	Aged 30 to 45 years old. All stages of disease. Undergoing active chemotherapy. Time since diagnosis: NR	Four themes: (a) psychological discomfort, (b) physical problems, (c) social dysfunction and (d) failure in the family role
Obeidat [37]	2013	Jordan	Experiences of information exchange following diagnosis of early stage BC	Semi-structured interviews (single). Content analysis	28	Aged 29 to 70 years old. All stages of disease. Mastectomy or lumpectomy < 6 months. Time since diagnosis: NR	Three themes: (a) knowledge about BC and its treatment, (b) communication of cancer diagnosis and treatment and (c) education on treatment side effects
Obeidat [38]	2013	Jordan	Personal meanings of diagnosis and surgical treatment for early stage BC	Semi-structured interviews (single). Heideggerian interpretive phenomenological analysis	28	Aged 29 to 70 years old. All stages of disease. Mastectomy or lumpectomy < 6 months. Time since diagnosis: NR	Four themes: (a) fearing BC, (b) trusting as coping, (c) surrendering to and suffering through treatment and (d) embodied self as changed One constitutive pattern: controlling fear
Saati [39]	2013	Saudi Arabia	Experiences of diagnosis and treatment	Semi-structured interviews (single) and focus groups. Grounded theory analysis	60	Mean age: 46 years old. Stage of disease: NR. All stages of treatment. Time since diagnosis: NR	Five themes: (a) communication, (b) acceptance, (c) knowledge and understanding, (d) limitations imposed by culture and (e) positive dimensions of culture
Soliman [40]	2018	Morocco	Experiences and perspectives of barriers to diagnosis and treatment of BC	Semi-structured interviews (single). Grounded theory analysis	25	Age: NR. Stage of disease: II to IV. All stages of treatment. Time since diagnosis: NR	Six themes: (a) treatment-associated costs, (b) burden of transportation and distance, (c) healthcare choice, (d) identity and femininity, (e) community influence and (f) spirituality and conception of death

*BC* breast cancer, *NR* not reported, *QOL* quality of life

### Synthesis results

Based on Arab women’s experiences of all stages of breast cancer three major themes were identified, ‘Perceptions and reactions’, ‘Coping or enduring’, and ‘Changing roles’, and seven sub-themes (Table 4).

**Table 4:** Themes and sub-themes

Theme	Sub-theme
(1) Perceptions and reactions	(1a) The diagnosis
	(1b) Perceptions of treatment
	(1c) Changing perceptions
(2) Coping or enduring	(2a) Challenges to coping
	(2b) Strategies for coping
(3) Changing roles	(3a) Care-provider versus care-receiver
	(3b) Positive new roles

#### Theme 1. Perceptions and reactions

##### 1a The diagnosis

The majority of diagnoses within the identified literature arose from women presenting with symptoms rather than through screening; however, many experienced a delay between first symptoms and receiving a diagnosis. This period was characterised by uncertainty and multiple visits to modern and traditional healthcare providers [23, 30, 39, 40]. The moment of receiving a diagnosis of breast cancer resulted in much fear and anxiety for Arab women [22–25, 31, 38, 40]. Nevertheless, they felt unable to openly express these concerns with their healthcare professionals. Instead, they passively followed the recommended investigations and treatments, or alternatively they sought second opinions and treatment elsewhere, including travelling abroad [23, 24, 34, 38, 39].

The subsequent trajectory of these reactions following the diagnosis diverged according to the healthcare setting. Women cared for in specialist centres felt well informed of their diagnosis and empowered in their treatment choices [24, 37]. In contrast, women who were cared for in local non-specialist hospitals often perceived that the healthcare professionals lied to them and provided incorrect management and advice [24, 30, 34, 37]. These experiences increased fear and mistrust which, at times, resulted in uncertainty of their diagnosis and a persistent perception of breast cancer as a death sentence [22, 24, 31, 32, 36–38, 40].

##### 1b Perceptions of treatment

The identified literature described Arab women’s experiences of a variety of cancer treatments such as radiotherapy, chemotherapy, mastectomy and lumpectomy. Women often perceived themselves as periphery to any treatment decisions, and occasionally underwent treatment against their expressed wishes [27, 32, 34, 37, 38]. Several authors labelled the described breast cancer treatments as losses. For example, women described losing their hair, a breast, a normal life, fertility, independence, autonomy, physical attractiveness and arm function [23, 24, 28–32, 36–38]. These losses, especially hair loss and mastectomy, were emphasised by respondents as negatively influencing their quality of life, physical well-being, body image and relationship with their husbands, if married [28, 32]. For some women, losing a breast was as equally shocking as learning of their diagnosis [36, 38]. However, over time, most came to accept their treatment-associated losses as minor in comparison to their diagnosis and their hope of a cure.

##### 1c Changing perceptions

Women’s perceptions of breast cancer changed as they progressed along their journeys with it. For many women, the early dominant view of breast cancer as a death sentence was replaced by the new concept of breast cancer being a curable illness or a chronic condition [29, 31, 32]. This process was described more often for those respondents who were several years post diagnosis; however, it also was evident in the experiences of women who had received options for treatment, and who had accessed information from the internet or from breast cancer survivors [23, 24, 28, 29, 31, 32, 34, 37, 38]. Nevertheless, breast cancer continued to be considered as a death sentence by women who remained uncertain of their diagnosis, feared recurrence or were anxious about passing the disease on to others in their family [23, 24, 28, 29, 32–34, 37, 38].

#### Theme 2. Coping with breast cancer

Arab women described coping with breast cancer as a continuous battle [29, 31]. They battled not just against cancer, but also against the stigmatisation they experienced.

##### 2a Challenges to coping

Arab women considered that their ability to cope with breast cancer was hindered by the cancer itself and by their family and society’s perception of the condition. Cancer- and care-related challenges included the financial burden of treatment, distressing symptoms such as pain, the fear of cancer recurrence, infertility, losing their independence and dying alone [23, 27, 29, 30, 33, 38, 40].

The responses of husbands and families were sometimes challenging for the respondents. Although husbands tended to be valued as sources of emotional and financial support, there were occasions where married Arab women were afraid that their husbands may divorce them or take a second wife, because they were no longer fertile or sexually attractive [25, 30, 32, 36]. There were infrequent examples in the literature where these fears were realised, with the rupture of sexual relationships, separation, divorce, and physical or emotional spousal abuse being experienced [24, 26, 30, 32, 35, 36].

Arab women found it difficult to cope with society’s negative reactions to their illness. These were characterised as being pitied, shunned and blamed for having breast cancer [25, 26, 29, 31, 33, 36, 38]. Respondents felt that many people, including friends and work colleagues, feared them and avoided them because of a fear that breast cancer was contagious. At times, a distrust of those around them was developed because of their experiences of being blamed for having breast cancer, a sign of punishment for undisclosed past sins or uncontrolled anger, or social humiliation and ridicule [22, 23, 26, 30, 32, 34]. Various strategies were therefore employed to cope with, or avoid, such distressing experiences.

##### 2b Strategies for coping

To avoid being viewed as a sick person, Arab women with breast cancer attempted to maintain their pre-diagnosis persona [25, 29, 31, 33, 38]. Several strategies were employed to either achieve this image, or to help when it was no longer possible for the women to maintain the portrayal as a well person. These were the non-disclosure of diagnosis, faith in Allah, receiving support from friends and family, and relationships with others with breast cancer.

*2bi Non-disclosure of diagnosis* Arab women preferred to conceal their diagnosis from others, at least initially. They managed this by not disclosing the diagnosis, hiding signs of illness or treatment side effects, and explicitly denying any problems; for example, wearing a wig or a traditional head covering (hijab) to hide hair loss [22, 24, 28, 30, 32, 33, 36, 38]. However, these attempts to conceal were often insufficient, such as during chemotherapy when the signs of illness were more visible. At these times, many women chose to isolate themselves from others [22, 23, 30, 36, 38]. While such responses tended to be temporary and treatment related, there were examples where prolonged isolation appeared to lead to low mood and depression for the women [22, 36, 38].

*2bii Faith in Allah* The Muslim faith played an important role in how Arab women coped with breast cancer. The belief and acceptance that life and death are predestined was a comfort to the women, because it meant that cancer, its treatment and its cure were controlled by Allah [23, 26, 27, 31, 38]. This acceptance of destiny was not fatalistic in nature and in contrast, women considered that they were equally obliged by religion to seek medical advice and comply with treatment.

Breast cancer was viewed as a test of faith, sent by Allah [22, 31, 39]. To pass this test, women perceived the need to respond to it with patience, endurance and acceptance [24, 31, 38, 39]. Exhibition of such responses would be eternally rewarded through the reduction of sins. Several older participants described their diagnosis as a gift because it had inspired a re-evaluation of what was important in life and the afterlife. However, the advantages of faith were not limited to the afterlife, as women considered that their faith in Allah helped them to remain hopeful for a cure and to endure the side effects of treatment [22, 23, 31, 38].

*2biii Receiving support from friends and family* The support of friends and family was valued by respondents. The nature of the help provided varied according to the relationship. For example, a husband’s role was to be the main provider of emotional and material support [22, 24, 28, 31, 34, 38, 39]. Friends, neighbours and the wider family were expected to visit the women’s homes to give moral support [22, 24, 27]. Although the women preferred to avoid the subject of cancer, they took comfort in being told stories of others being cured of cancer [20]. In contrast, female members of the immediate family provided practical support and help, such as helping with the household chores and child care [24, 28, 31, 38]. Financial aid was often received from the wider family, even if there was no great need [24, 34].

*2biv Relationships with other women with breast cancer* Contact with other women with breast cancer helped the women cope with their own diagnosis. Firstly, participants appreciated knowing that they were not alone in their experiences [22, 26, 32]. Secondly, they made reassuring comparisons between themselves and others who were less fortunate, for example, those with more advanced disease or those who were young and unmarried [24, 28, 34, 38]. Thirdly, there were examples where women, post treatment and in good health, felt empowered and encouraged to support others with breast cancer, especially those more recently diagnosed [24, 28, 29, 32].

#### Theme 3. Breast cancer and roles

The role of women in the Arab societies was primarily viewed as being a daughter, a wife or a mother. However, a diagnosis of breast cancer threatened these identities and roles [25, 31, 32, 36, 38, 40].

##### 3a Care-provider versus care-receiver

The principal priority for many Arab women was to protect and promote their family’s well-being. Women initially concealed their diagnosis so as to protect others from distress and sadness, especially their children [32, 38]. They therefore experienced frustration and sadness when breast cancer rendered them care-receivers, in contrast to being self-sufficient care-providers. This switching of roles was especially evident during active treatment or with an increasing disease burden [31, 36, 38].

Arab women’s role of care-provider was not limited to practical tasks; they were also partly responsible for the family’s social and spiritual well-being. It was therefore distressing for those women who blamed themselves for introducing breast cancer into the family [29, 30, 34]. They considered that they failed at their role because they could no longer provide the best chances for their family, which included the nurturing of marriage opportunities for their unmarried female relatives [29, 30, 34].

##### 3b Positive new roles

For many women, a diagnosis of breast cancer stimulated a re-evaluation of what they valued in life, which leads to new post-diagnosis identities and roles [26, 32, 38]. Women challenged society’s view of them as sick women waiting for death to take them, by encouraging the view that breast cancer was a curable or chronic illness, equal or even favourable to diabetes [29, 31]. New positive roles included being respected informal advisors to other women with breast cancer, or strong women able to endure and accept their destiny [22, 28, 29, 38]. These new identities strengthened the argument against society’s stigmatisation of them [30, 38].

In contrast to seeking a new role in society, there were occasions where Arab women with breast cancer rejected the idea of re-entry into society’s roles and normality. Instead, they embraced the idea of breast cancer as a death sentence and were keen to demonstrate their acceptance before Allah and others [22, 24, 31]. They argued that death was the inevitable outcome for all, irrespective of whether they have cancer or not [22, 26].

## Discussion

This thematic synthesis of qualitative data has generated an understanding of how breast cancer is experienced by Arab women in Arab countries. The broad range of literature identified and synthesised in this review, representing 401 women in 11 different countries, has illustrated both convergences and divergences of experiences and impact upon their reported HRQOL. This review has illustrated how Arab women’s experiences are formed in response to their perceptions of cancer and its evolution within an interplay of individual, social and religious factors.

At the personal level, the identified literature suggests that much of Arab women’s negative experiences, such as anxiety, fear, and symptoms, are most problematic around the time of diagnosis and during active treatment. This is consistent with the broader literature, which suggests that breast cancer survivors go on to have a good quality of life [8, 41]. However, Arab women who remain symptomatic and uncertain of their diagnosis with limited access to reliable information may struggle to transition to the ideal new identity as strong breast cancer survivors, even several years post treatment. While many of these fears and worries are consistent with other populations, such as the fear of recurrence [42, 43], other concerns suggest a closer association with the Arab context, such as the avoidance of pity, and the transition from care-provider to care-receiver.

An appreciation of how Arab women perceive their role in their social context is helpful to understand and interpret their experiences. They identify as mothers, wives and daughters, and their role is to provide and protect their family, sacrificially if required. Therefore, many of the experiences of breast cancer are challenging for them because of their impact on their family rather than on themselves. For example, the initial response to a diagnosis is to conceal it and protect others from emotional upset. Arab women were afraid that their diagnosis of breast cancer had damaged and tarnished their family. This was understood as either through hereditary means or infectious contagion. For women with an awareness of breast cancer genetics, many consider that they have introduced breast cancer into their family blood-line and are thereby guilty for reducing the marriage chances for their daughters and grand-daughters. This fear may be intensified and more common in this ethnic group because of the frequent practice of the family vetting potential marriage partners for suitability, fertility and character [29, 30]. In addition, some Arab countries have a mandatory pre-marital genetic testing due to the high rates of consanguineous marriage and the increased transmission rates of autosomal recessive disorders, such as β-thalassemia [44]. In contrast, women with limited access to information perhaps focus more on their fear that breast cancer is contagious, and thus perceive themselves to be a risk to their family’s physical well-being. It is pertinent to note that these fears are founded on a shared perception that they and their family risk being stigmatised because of their diagnosis. Consequently, women promote breast cancer as a normal disease with a cure to counter such stigmatisation. Alternatively, some women embrace their mortality and encourage others to do likewise reinforcing the Islamic view that it is Allah who is fully in control of the manner, moment and place of their death, and not breast cancer.

Islam has been a major influence in Arab countries since the eleventh century, although there is currently much blurring of the boundaries between religion and culture in the region [45]. Nevertheless, the practices and interpretations of Islam are perceived as defining much of how health and illness are experienced, for example encouraging adherents to interpret breast cancer as a divine test of faith [46]. Such a view is not unique to the Arab context, and is a common experience for non-Arab Muslims and other religious groups [43, 47, 48]; however, it is important to give credence to the importance that Arab women attribute to their faith and the felt need to demonstrate polite appreciation to Allah in all circumstances. In light of this, they consider that there are potential advantages to having breast cancer, both for this life and the next. For example, in this life, women hold that an appropriate response to breast cancer may lead to a reprioritisation of their life values, improved relationships with husbands, friends and family, and eternal rewards from Allah, in the next life. Errihani et al. [49] recognise a duality of effectiveness in this approach. They suggest that, for Muslim patients, increased religious practices can, firstly, increase chances of a cure because it is Allah who controls the effectiveness of any treatment; and secondly, even if religion does not help them to recover, it may help them enter paradise after their death.

### Strengths of the literature review

This review was strengthened by the breadth of the articles identified. Included data in this review represented 11 heterogeneous Arab countries, with much variation in their cancer services. For example, the five-year breast cancer survival in Jordan, with an annual health expenditure of $224 per capita, is 43.1% in contrast to 78.4% in Saudi Arabia which expends $1147 [50]. Such heterogeneity was compounded by the inclusion of rural and urban populations, high- and low-income families, all stages of breast cancer and all stages of treatment, including pre-treatment, active treatment and survivorship.

### Limitations of the literature review

There were several limitations of the literature review. Firstly, the heterogeneity of the data while providing rich diverse descriptions of Arab women’s experiences may also hinder the understanding of experiences across this geographical and ethnic region and within sub-groups of the population. Secondly, as with any review, it was limited by the available data identified. The 11 countries represented in this review tend to be higher income and therefore the voices of Arab women with breast cancer from middle- and low-income Arab nations feature less in this review. Subsequently, there continues to be a need for further research exploring the experiences and HRQOL of women with breast cancer in Arab region. This should include primary qualitative research in addition to an updated, broader systematic review of the quantitative data exploring HRQOL in women with breast cancer in the Arab region.

## Conclusion

The purpose of this literature review was to understand women’s experiences of breast cancer in Arab countries, and its impact upon their quality of life. The review concludes that the experiences of Arab women with breast cancer are strongly influenced by their family, social, religious and healthcare contexts. Many of these experiences are negative and distressing. Women balance their belief and hope that breast cancer comes from Allah while questioning the veracity of society’s assumption that breast cancer is a death sentence. The journeys of women, who are actively implicated in decision making, have access to appropriate information, and receive support from husbands, family and friends, appear to follow similar trajectories towards survivorship as described in higher income settings. In contrast, Arab women tend to remain uncertain and fearful when they have limited access to reliable information or treatment choices. Further research comparing these findings with women elsewhere would be useful. A person-centred approach should therefore be encouraged, in which Arab women are provided with appropriate information in accordance to their understanding and desires.

## Electronic supplementary material

Below is the link to the electronic supplementary material.

Supplementary material 1 (DOCX 12 kb)
