# Supplementary material for: Experiences of breast cancer in Arab countries. A thematic synthesis
Source: Qual Life Res. 2019 Oct 23;29(2):313–24. doi: 10.1007/s11136-019-02328-0 (PMC6994422; doi:10.1007/s11136-019-02328-0)
Supplement: Supplementary file 1 — Supplementary material 1 (DOCX 12 kb) [file 11136_2019_2328_MOESM1_ESM.docx]

Supplementary file

PubMed Search (21 March 2019)

|  | **Term** | **Results** |
| --- | --- | --- |
| 1 | Breast OR Mammary [ALL FIELDS] | 461,854 |
| 2 | Cancer OR Tumour OR Tumor OR Malignan* OR Neoplasm [ALL FIELDS] | 3,794,632 |
| 3 | Breast Neoplasms [MeSH Terms] | 243,202 |
| 4 | Arab OR Algeria* OR Bahrain* OR Comoros OR Comori* OR Djibouti* OR Egypt* OR Iraq* OR Jordan* OR Kuwait* OR Leban* OR Libya* OR Mauritania* OR Morocc* OR Oman* OR Palestin* OR Qatar* OR Saudi Arabia* OR Somalia* OR Sudan* OR Syria OR Tunisia* OR United Arab Emirates OR Emirat* OR Yemen* [ALL FIELDS] | 235,576 |
| 5 | Adaptation OR Coping OR Enduring OR Lived experience OR Perception* OR Experience* OR Perspective* OR View* OR Idea* OR Expectation* OR Belief* OR Believe* OR Motivat* OR barrier* OR Narrative OR Survivor* OR Attitudes [ALL FIELDS] | 2,808,301 |
| 6 | Depression OR Psychological OR Quality of life OR Perception OR Social support OR Risk assessment OR Cultural characteristics OR Health knowledge, attitudes, practice OR Survivors/Psychology OR Anxiety [MeSH Terms] | 1,940,138 |
| 7 | 1 AND 2 | 358,627 |
| 8 | 3 OR 7 | 357,370 |
| 9 | 5 OR 6 | 3,936,473 |
| 10 | 4 AND 8 AND 9 | 1025 |
